# Carotidynia—A Rare Cause of Anterior Neck Pain: Case Report

**DOI:** 10.5811/cpcem.48896

**Published:** 2026-01-20

**Authors:** Faith Ibu, Olivia Keller-Baruch, Jessica Pelletier

**Affiliations:** * Mayo Clinic, Department of Emergency Medicine, Rochester, Minnesota; † Ichilov Medical Center, Department of Emergency Medicine, Tel-Aviv, Israel; ‡ University of Missouri-Columbia, Department of Emergency Medicine, Columbia, Missouri

**Keywords:** carotidynia, neck pain, carotid inflammation, case report

## Abstract

**Introduction:**

Carotidynia is a rare, often under-diagnosed condition characterized by idiopathic inflammation around the carotid artery, presenting with unilateral neck pain that typically resolves within two weeks.

**Case Report:**

A 32-year-old male presented with intermittent right anterior neck pain for two years, with no other associated symptoms. Computed tomography revealed carotid perivascular inflammation, consistent with carotidynia.

**Conclusion:**

Although self-limited, carotidynia should be considered in patients with unexplained neck pain, as its recognition is crucial to differentiate it from more serious conditions and to avoid mismanagement or unnecessary interventions.

## INTRODUCTION

Carotidynia first emerged as a medical term in the late 20th century and has since been attributed varying definitions and diagnostic criteria, leading to confusion and controversy within the medical community.^1^ In 1988, carotidynia was categorized by the International Classification of Headache Disorders as an atypical headache syndrome separate from idiopathic neck pain^2^; the diagnosis required unilateral neck pain, focal carotid tenderness, absence of structural lesions, and spontaneous resolution within 14 days for diagnosis.^3^ However, in 2004, carotidynia was excluded from the classification system due to concerns over diagnostic validity, and it was redefined simply as a nonspecific symptom of other diseases.^4^ More recently, however, there has been recognition of characteristic radiologic findings that favor carotidynia as a distinct entity, now more commonly referred to as transient perivascular inflammation of the carotid artery (TIPIC) syndrome.^3^,^5^ While it remains a rare disorder, reported cases suggest a slight female predominance, with peak incidence in the fifth and sixth decades of life.^5^ The disease is thought to be driven by an underlying transient inflammatory process of the carotid adventitia, although its exact pathogenesis remains debated.

## CASE REPORT

A 32-year-old male presented to the emergency department (ED) with acute exacerbation of chronic right anterior neck pain. He reported that the pain had been intermittent for approximately two years. He reported severe pain in the same area, lateral to the hyoid bone on the right. He denied fevers or infectious symptoms around the onset of the pain or during any recurrences. He reported exacerbation of pain with leaning forward and sometimes lying flat to sleep, with no change with swallowing. He denied any systemic symptoms such as fever, dysphagia, trismus, neurologic, respiratory, or gastrointestinal complaints. He had taken acetaminophen and ibuprofen with only temporary relief. The current flare-up began five days prior and had not improved despite taking acetaminophen, ibuprofen, amoxicillin, and acetaminophen-hydrocodone, which he had available at home.

He was seen in the ED the previous day, diagnosed with suspected hyoid-related musculoskeletal pain, and discharged after declining osteopathic manipulation and additional analgesics. His pain had worsened, prompting his second ED visit. In the ED, vitals were within normal limits, and he was overall well-appearing. Physical examination revealed no swelling or asymmetry, marked tenderness to palpation over the right anterior neck around the area overlying the common carotid bifurcation. He had no abnormal neurological findings. There was no pharyngeal erythema or swelling, or evidence of dental infection, and the physical examination was otherwise non-contributory.

**Image 1:**
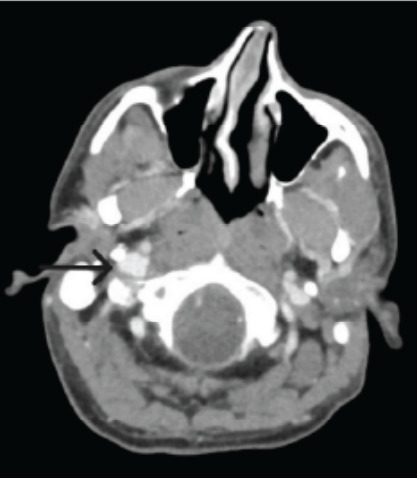
Axial computed tomography with a black arrow showing mild, asymmetric circumferential inflammation of the right carotid and extracranial internal carotid arteries.

**Image 2:**
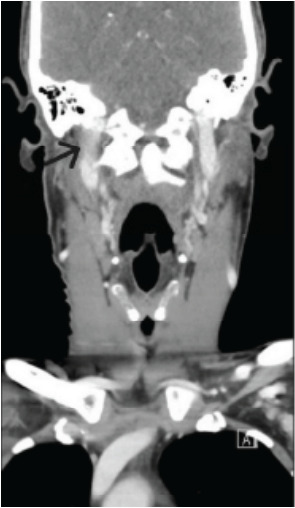
Coronal computed tomography with a black arrow demonstrating mild, asymmetric circumferential inflammation of the right carotid and extracranial internal carotid arteries.

Lab workup was within normal limits, including complete blood count and inflammatory markers, making an acute infectious process less likely. A computed tomography scan of the soft tissues of the neck with intravenous contrast demonstrated circumferential inflammation of the right carotid and extracranial internal carotid arteries (Images 1 and 2), correlating with the patient’s region of tenderness.

Given the patient’s clinical presentation in concordance with these characteristic imaging findings, a diagnosis of carotidynia was made. The patient was counseled on the diagnosis and likely disease course and was treated with scheduled nonsteroidal anti-inflammatory drugs (NSAID) and a short course of steroids. A follow-up appointment was scheduled with vascular surgery. On follow-up about two months later, he reported complete resolution of symptoms. He had a CT angiography and a carotid ultrasound with resolution of prior findings. No additional interventions were provided, and the patient was counseled to follow up as needed for recurrent symptoms.

*CPC-EM Capsule*

What do we already know about this clinical entity?

*Carotidynia is a rare, self-limiting condition characterized by idiopathic inflammation around the carotid artery that more often occurs in middle-aged women.*

What makes this presentation of disease reportable?

*We present the case of a young male diagnosed with this condition who was successfully treated with Nonsteroidal Anti-Inflammatory Drugs.*

What is the major learning point?

*While the exact etiology of carotidynia remains obscure, it presents with distinct radiologic findings that allow for accurate diagnosis.*

How might this improve emergency medicine practice?

*Recognizing carotidynia enables the clinician to distinguish it from more serious pathologies and to prevent unnecessary interventions and potential repeat visits.*

## DISCUSSION

Carotidynia is a rare clinical entity with a reported prevalence of about 2.8% of patients presenting with acute neck pain in one study.^5^ Over the years, since its first description in 1927, there has been controversy over this diagnosis, further adding to its obscurity.^1^,^3^,^5^ Nonetheless, its characteristic radiographic findings suggest it to be a distinct clinical entity. As in this case, most patients report sudden-onset, throbbing, unilateral neck pain that may radiate to the jaw, ear, or ipsilateral eye and may be exacerbated with swallowing or head movement. Patients may also report bilateral symptoms and recurrent, self-resolving episodes.^5^ On physical examination, the cardinal finding is exquisite focal tenderness upon palpation of the carotid artery bifurcation.^5^

In most studies, no neurologic symptoms have been reported; however, Lecler et al reported varying transient neurological symptoms such as dizziness, vertical diplopia, facial nerve palsy, dysesthesia, and motor deficit in about 17% of patients (eight of 47).^5^ Differential diagnosis is critical in managing carotidynia due to its symptom overlap with other conditions; it involves considering the patient’s entire clinical picture, including history and symptom progression. Carotid artery dissection, for instance, presents with neck pain and tenderness but often includes focal neurological symptoms, which are absent in carotidynia.^6^ Similarly, thyroiditis may cause neck pain but is accompanied by systemic symptoms and thyroid dysfunction, which can be distinguished through lab tests.^7^

Imaging proves invaluable in differentiating carotidynia or TIPIC syndrome from these conditions. On ultrasound (US), carotidynia presents with hypoechoic wall thickening at the region of tenderness along the carotid artery with associated mild luminal narrowing.^3^,^5^ Ultrasound can also detect flow disruption or stenosis, which is not expected in carotidynia.^3^ The perivascular thickening around the carotid artery that is characteristic of the condition can also be distinguished on both CT and magnetic resonance imaging (MRI).^5^ Additionally, these imaging modalities can aid in visualizing vascular integrity and ruling out structural anomalies. The exclusion of these other potential causes is essential to arrive at a diagnosis of carotidynia, guiding appropriate treatment and preventing unnecessary interventions.

Regardless of modality (US, CT, MRI), typical imaging features of carotidynia include eccentric thickening and enhancement of the wall of the distal common carotid artery, bulb and proximal internal carotid artery, mild luminal narrowing, and fat-stranding in surrounding tissues.^5^ Unfortunately, the sensitivity and specificity of imaging studies for diagnosing carotidynia have not been established in the literature, and there is no validated gold standard modality for diagnosis. It is important to note that, like the self-limiting nature of the symptoms associated with the disease, the imaging abnormalities are also transient in nature, further complicating the diagnostic process.^5^

In the past, antimigraine medications such as propranolol, tricyclic antidepressants, ergotamine, and methysergide were used for the treatment of carotidynia.^8^ However, this trend decreased once carotidynia was removed from the International Classification of Headache Disorders in 2004. Now, given that most cases self-resolve, treatment typically includes rest, reassurance, and NSAIDs or aspirin. Symptom resolution is expected within one to two weeks. Additional treatments like corticosteroids, low-dose benzodiazepines, and calcium channel blockers have shown some success in recurrent cases.^9^

## CONCLUSION

In emergency medicine, the ability to rapidly identify and differentiate carotidynia from other potentially life-threatening conditions is paramount. Imaging modalities such as CT, ultrasound, and MRI can be used to visualize the integrity of the carotid artery and surrounding structures. While carotidynia is a self-limiting disease, other causes of unilateral neck pain that are more severe and may even be life-threatening, such as carotid dissection, thyroiditis, giant cell arteritis, sialadenitis, or cervical arthrosis, must first be excluded. The role of imaging in carotidynia extends beyond diagnosis, contributing to a deeper understanding of its inflammatory nature and helping guide clinical management to ensure timely and effective treatment.
